# Supplementary material for: Curated and harmonised transcriptomics datasets of interstitial lung diseases
Source: Data Brief. 2025 Oct 14;63:112139. doi: 10.1016/j.dib.2025.112139 (PMC12581653; doi:10.1016/j.dib.2025.112139)
Supplement: Supplementary file 1 [file mmc1.zip › Supplementary_material/RNA-seq/fastQC reports/GSE184316/SRR15926739_fastqc.html]

SRR15926739.fastq.gz FastQC Report 

FastQC Report

Mon 5 Feb 2024  
SRR15926739.fastq.gz

## Summary

- Basic Statistics
- Per base sequence quality
- Per sequence quality scores
- Per base sequence content
- Per sequence GC content
- Per base N content
- Sequence Length Distribution
- Sequence Duplication Levels
- Overrepresented sequences
- Adapter Content

## Basic Statistics

| Measure | Value |
| --- | --- |
| Filename | SRR15926739.fastq.gz |
| File type | Conventional base calls |
| Encoding | Sanger / Illumina 1.9 |
| Total Sequences | 9136675 |
| Sequences flagged as poor quality | 0 |
| Sequence length | 25-384 |
| %GC | 51 |

## Per base sequence quality

## Per sequence quality scores

## Per base sequence content

## Per sequence GC content

## Per base N content

## Sequence Length Distribution

## Sequence Duplication Levels

## Overrepresented sequences

| Sequence | Count | Percentage | Possible Source |
| --- | --- | --- | --- |
| CACCTCCCCGAAGCTCAGGGAGAGCCCTGTTAGGGCCGCCTCTGGCCCTA | 59212 | 0.6480694563394233 | No Hit |
| CAATCCACTTGATCCCAACTCATCTCTCATTTATTTCGGCTTCTTTTATT | 58633 | 0.6417323588723469 | No Hit |
| CAGTGGGGCCACGAGCTGAGTGCGTCCTGTCACTCCACTCCCATGTCCCT | 58248 | 0.6375185721282632 | No Hit |
| TTACAAAAGCAGAATAAAAGCGAAAAGAAATGAAAATGTTACACTACATT | 45371 | 0.4965810866644595 | No Hit |
| AGGCTACACACTGAGGACTCTGTTCCTCCCCTTTCCGCCTAGGGGAAAGT | 38135 | 0.4173837856769558 | No Hit |
| AGACATCAAATCCTGCAGACAAGGGGAGCCCTCAGTCTGCAGGGCTCCAT | 33973 | 0.37183110923831697 | No Hit |
| GGGAAGCGGGGAATGTCTACGTGCGTATGCACGTGGCACTCTCTGCCCGA | 29978 | 0.32810623120555343 | No Hit |
| GGCATCAAAAGTGATGGACTGCCCATTGCTGGAGAAGACCTTCTCTCCTA | 28856 | 0.3158260526942241 | No Hit |
| TGATATGGTTCCTGGCAAGCCCATGTGTGTTGAGAGCTTCTCAGACTATC | 28827 | 0.3155086505758386 | No Hit |
| CTTCATGATGTAGCAGCAGGTGCCAGGGGCTGGCTTGTAGGCGATCAGCA | 28765 | 0.31483006673653163 | No Hit |
| TACCACTGCCACCTTCTCCATCGGCTCCACTGGCCTCGTGGTGTATGACT | 26076 | 0.2853992289317503 | No Hit |
| TGATGACACCCACCGCAACTGTCTGTCTCATATCACGAACAGCAAAGCGA | 24720 | 0.27055794367206887 | No Hit |
| GGCAATGAGCGGTTCCGCTGCCCTGAGGCACTCTTCCAGCCTTCCTTCCT | 22282 | 0.24387427592641744 | No Hit |
| GTCCACGTCACACTTCATGATGGAGTTGAAGGTAGTTTCGTGGATGCCAC | 21583 | 0.23622379038326305 | No Hit |
| ACAAGAAGTACATCAAAGATTACATGAAATCAATCAAAGGGAAACTTGAA | 14977 | 0.16392177679516892 | No Hit |
| CTCTCTTTCTGGCCTGGAGGCTATCCAGCGTACTCCAAAGATTCAGGTTT | 14972 | 0.16386705229199902 | No Hit |
| ATCAAAGAAGAACATGTGATCATCCAGGCCGAGTTCTATCTGAATCCTGA | 14962 | 0.16375760328565916 | No Hit |
| TTCAGCCCTGATCAAGACATGAGGGAGGCAGGGGCTCAGCTGAAGAAGCT | 14551 | 0.159259249125092 | No Hit |
| GAAACCCAGACACATAGCAATTCAGGAAATTTGACTTTCCATTCTCTGCT | 13838 | 0.15145553497306188 | No Hit |
| ACGTTTACCTGTCCATGTCTTACTACTTTGACCGCGATGATGTGGCTTTG | 13795 | 0.15098490424580058 | No Hit |
| ACCTCTCTCTGGGCTTCTATTTCGACCGCGATGATGTGGCTCTGGAAGGC | 13648 | 0.14937600385260502 | No Hit |
| CAATGGGGTACTTCAGGGTCAGGATGCCACGCTTGCTCTGGGCCTCGTCG | 12450 | 0.13626401289309295 | No Hit |
| CAGCATGTTCCCTCTCCTCATGAGATTGGTGAAGAAAGTATTTGGCAAAG | 12346 | 0.13512574322715867 | No Hit |
| CCCTGGCTGCTACAGGAGAATAGCAGACAGGTATAGTTAAGAAACAACTT | 12121 | 0.13266314058451242 | No Hit |
| AGCTGCTAAGTGCTGTGTTGTCGTTCCCCCTGCTTAAAATAAAGTTGTTT | 12046 | 0.13184227303696366 | No Hit |
| GTTCTGCAGCCCCTGTCATAAAAGGTTTTACTCTTTCTGGTCTCTGTTCT | 10835 | 0.1185879983692098 | No Hit |
| TTTTTCCATGAGCTTAATGATGCTTTCTCTGGGCTTTTGGGGGAGGGTGT | 10684 | 0.11693531837347831 | No Hit |
| CGATCAGCACGGGCTCGTACTCTGCCACAAACTGATCACACTGCTTCTGG | 9955 | 0.10895648581130445 | No Hit |
| CAGCAGTTTACACCTGCTAGGGTGTTCAAAGGTCAGTGCTATAGAAATTC | 9515 | 0.10414072953235176 | No Hit |
| GGCAATGAGCGGTTCCGGTGTCCGGAGGCGCTGTTCCAGCCTTCCTTCCT | 9415 | 0.10304623946895343 | No Hit |

## Adapter Content

Produced by FastQC (version 0.11.7)
